# “First, do no harm”: legal guidelines for health programmes affecting adolescents aged 10–17 who sell sex or inject drugs

**DOI:** 10.7448/IAS.18.2.19437

**Published:** 2015-02-26

**Authors:** Brendan Conner

**Affiliations:** Technical Advisor, HIV Young Leaders Fund

**Keywords:** young key populations, adolescent key populations, young drug users, harm reduction, commercial sexual exploitation of children, sexually exploited children, juvenile prostitution, young sex workers, young people, minimum intervention

## Abstract

**Introduction:**

There is a strong evidence base that the stigma, discrimination and criminalization affecting adolescent key populations (KPs) aged 10–17 is intensified due to domestic and international legal constructs that rely on law-enforcement-based interventions dependent upon arrest, pre-trial detention, incarceration and compulsory “rehabilitation” in institutional placement. While there exists evidence and rights-based technical guidelines for interventions among older cohorts, these guidelines have not yet been embraced by international public health actors for fear that international law applies different standards to adolescents aged 10–17 who engage in behaviours such as selling sex or injecting drugs.

**Discussion:**

As a matter of international human rights, health, juvenile justice and child protection law, interventions among adolescent KPs aged 10–17 must not involve arrest, prosecution or detention of any kind. It is imperative that interventions not rely on law enforcement, but instead low-threshold, voluntary services, shelter and support, utilizing peer-based outreach as much as possible. These services must be mobile and accessible, and permit alternatives to parental consent for the provision of life-saving support, including HIV testing, treatment and care, needle and syringe programmes, opioid substitution therapy, safe abortions, antiretroviral therapy and gender-affirming care and hormone treatment for transgender adolescents. To ensure enrolment in services, international guidance indicates that informed consent and confidentiality must be ensured, including by waiver of parental consent requirements. To remove the disincentive to health practitioners and researchers to engaging with adolescent KPs aged 10–17 government agencies and ethical review boards are advised to exempt or grant waivers for mandatory reporting. In the event that, in violation of international law and guidance, authorities seek to involuntarily place adolescent KPs in institutions, they are entitled to judicial process. Legal guidelines also provide that these adolescents have influence over their placement, access to legal counsel to challenge the conditions of their detention and regular visitation from peers, friends and family, and that all facilities be subject to frequent and periodic review by independent agencies, including community-based groups led by KPs.

**Conclusions:**

Controlling international law specifies that protective interventions among KPs aged 10–17 must not only include low-threshold, voluntary services but also “protect” adolescent KPs from the harms attendant to law-enforcement-based interventions. Going forward, health practitioners must honour the right to health by adjusting programmes according to principles of minimum intervention, due process and proportionality, and duly limit juvenile justice and child protection involvement as a measure of last resort, if any.

## Introduction

It is well settled that adolescent key populations (KPs) face heightened health risks as a result of law and policy barriers to accessing HIV treatment, diagnostic and prevention services and that the stigma, discrimination and criminalization experienced by adolescent KPs aged 10–17 is intensified, as compared to their older cohorts. Those adolescents who sell sex or inject drugs more often face aggravating circumstances such as family rejection and street-involvement, combined with legal constructs concerning consent and age of majority. These constructs may condition access to life-saving treatment on parental consent or proof of “emancipation,” or lower the age of consent to sell sex or use drugs as compared to general consent-to-sex law, resulting in increased police encounters and commitment to state custody [1,2].

This commentary addresses only adolescent KPs aged 10–17 and not adolescents aged 18–19 in order to spotlight the specific, legal guidelines for health and protective interventions that apply to persons under the legal age of majority. It must be cautioned that developmentally adolescence is a dynamic state and that major differences may exist in identity, understanding and behaviour between adolescents within this range. While it is true that adolescents aged 10–17 who are transgender (TG) or young men who have sex with men (YMSM) face related challenges [3,4], in order to tailor recommendations to the special legal framework applicable to these populations, this paper narrows its scope to adolescents who sell sex or use drugs, including the disproportionate number of adolescent TG and YMSM who do so.

There is a strong and emergent evidence base that law enforcement-based interventions targeting adolescents aged 10–17 who sell sex or use drugs result in affirmative harm to the very same adolescents they are intended to protect [1,2,5–7]. The daily reality reported by many adolescents who sell sex or use drugs is dominated by harassment, theft, detention, deportation, and physical and sexual violence by law enforcement and military personnel, including rape and extortion in exchange for release [1,2,8–10]. This lived experience of violence and corruption instills fear in adolescents and prevents adolescent victims from reporting the crimes committed against them [1,2,8]. It similarly poisons relationships with service providers who may be compelled to report adolescents to law enforcement as a result of mandatory reporting laws [1,2]. What is more, adolescent KPs report abuse by health practitioners themselves, including discrimination and service denial, and even physical and sexual violence, forced abortions, breach of confidentiality and mandatory HIV testing [1,2,8].

Despite this frightening reality, a child protection framework is frequently applied at the country level to justify arrest-based interventions without reference to international human rights law governing the administration of juvenile justice, child protection and the right to health. The overwhelming majority of low- and middle-income countries possess little to no specialized child protection services. Thus “child protection” falls to precisely those persons identified by young KPs as perpetrators of violence: uniformed services, primarily police and military personnel. Even in high-income countries, arrest-based interventions are relied upon, with diversion to “services,” if at all, only *after* arrest in violation of international law and without clear guidelines for appeal or periodic review of the conditions of confinement [1,8,11]. Ironically, the very same interventions championed in the name of “child protection” are therefore increasing the numbers of one such KP – adolescents in prisons and other closed settings.

The confusing policy and programme environment has caused many healthcare providers to limit or cease services to adolescents aged 10–17 who sell sex or use drugs. In effect, implementation of international legal principles has drawn a red line at the age of majority that health practitioners dare not cross. The strong, evidence-based technical guidelines that exist for rights-based health interventions among sex workers and injecting drug users *over* the age of majority decline to extend rights-based recommendations to KPs aged 10–17 even when medical science invites an equivalent approach [12,13]. This commentary calls for a reexamination of the treaty framework, situating child-protective interventions firmly within international law and guidance governing the right to health, and regulating juvenile justice and child welfare interventions according to principles of minimum intervention, last resort, due process and proportionality. These important principles clarify that state obligations to respect, protect and fulfil the rights of KPs aged 10–17 are not simply directed at the prohibition of behaviours, but also to protect vulnerable young people from the inherent harms of certain law enforcement-based interventions undertaken for the purposes of “protection,” which are a direct result of the current misreading of the international legal framework.

The commentary also advances concrete guidelines for framing health programme interventions, specifying that:

1. The principle of non-criminalization mandates non-compliance of healthcare providers with arrest-based interventions, an immediate end to arrest and prosecution of adolescent KPs aged 10–17, and the abolition of involuntary custodial placement in the name of “rehabilitation”;
2. Voluntary, confidential and adolescent-friendly primary, sexual and reproductive health services;
3. The right of adolescents aged 10–17 who sell sex or use drugs to be heard includes meaningful participation in policy and decision-making in health services and other programmes that concern them, as well as reliable complaint procedures and remedies for rights violations;
4. Parental consent waiver for life-saving sexual and reproductive health services, HIV and harm-reductionist treatment;
5. Client-centred informed consent and right to refuse or consent to participation in medical treatment and research trials.

## Discussion

The Convention on the Rights of the Child is organized around the principle that “[i]n all actions concerning children … the best interests of the child shall be a primary consideration” [14, art. 3(1)]. This principle expressly includes non-state actors such as civil society groups and medical practitioners whose actions concern children [14]. This determination depends on a variety of individual circumstances, such as the nature of the decision being made, the age and the level of maturity of the child, the views of the child, the capacity and circumstances of caregivers to provide adequate food, clothing and medical care, and the safety and health risks of the alternative circumstances proposed. As such the proposed programmatic guidelines must be adjusted to the individual circumstances of the adolescent. Nonetheless, this commentary advances several preliminary guidelines based on international law, with the expectation that international agencies and civil society groups led by those adolescents affected will revisit them.

### 

#### The principle of non-criminalization

The categories “respect, protect and fulfil” are often used to summarize the obligations of States parties as signatories to human rights treaties. States parties are obliged to “respect” by not interfering with the enjoyment of human rights, to “protect” individuals and groups against human rights abuses and to “fulfil” by taking positive action to facilitate the enjoyment of basic human rights [15]. In reference to adolescents aged 10–17 who sell sex or use drugs, the obligation to “protect” has suffered from a perverse misapplication at the country level. The child protection framework has been used to justify law enforcement-based interventions without reference to international human rights law governing the administration of juvenile justice, child protection and the right to health.

The four guiding principles identified by the CRC Committee include non-discrimination (article 2); devotion to the best interests of the child (article 3); the right to life, survival and development (article 6); and respect for the views of the child (article 12) [16]. With the exception of the non-discrimination principle, these guidelines largely rely on an understanding of adolescents’ “positive” rights, as in an adolescent's right *to* receive life-saving medical treatment or right *to* have her view respected. Yet in prioritizing these principles, the Committee neglects the Convention's protection of “negative” liberties, primarily those international standards governing the application of judicial measures, institutional placements and protective interventions targeting persons under the age of majority. The CRC Committee has repeatedly ruled that no provision of the Convention may be read in isolation from other provisions more conducive to the rights of the child [17,18].

This neglect of the Convention's principles of negative liberty likely stems from the Convention's article 33 and 34 stipulations that States parties must “take all appropriate measures … to protect children from the use of narcotic drugs and psychotropic substances” and to “protect the child from all forms of sexual exploitation …” [14, arts. 33–34]. The Optional Protocol to the Convention on the Rights of the Child on the Sale of Children, Child Prostitution and Child Pornography (OPSC) also requires States parties to adopt criminal, civil and administrative penalties for the sale of children, child prostitution and child pornography [19]. The Protocol also expands the Convention's guarantee of protection of the rights and interests of child victims in criminal proceedings against their perpetrators [19]. The initial difficulty is that these treaties remain silent on the definition of “appropriate measures” for protective interventions. The qualifier “appropriate” is said to have been intended to act as a prophylactic against “arbitrariness, disproportionate measures and abuses of human rights in pursuit of protecting children ….” [7, p. 37, ¶ 73]. Commentators have interpreted the term to indicate that protective interventions must be based on adequate data, targeted and effective and proportionate such that they are in pursuit of a legitimate aim and tailored such that they are no more than necessary for the achievement of that aim [7, pp. 38–39, ¶ 76].

While a comprehensive framework regulating protective measures has eluded the CRC Committee, it has articulated certain limits. The CRC Committee has stated in the context of adolescents aged 10–17 who sell sex that the obligation to protect extends to ending their arrest and prosecution under national criminal or other laws [20]. The CRC Committee has consistently noted in its dialogue with States parties to the Optional Protocol that those adolescents who sell sex should “be neither criminalized nor penalized, and that all possible measures should be taken to avoid their stigmatization and social marginalization” [21, p. 8, ¶ 25]. The Committee has specifically criticized States parties with inadequate legislation and contradictory provisions on this issue [20,21]. The jurisprudence on arrest-based interventions for adolescents aged 10–17 who use drugs is slightly less certain, although the Committee's Concluding Observations in reference to article 33 protection from drug use and dependence use similar phrasing, namely that a child who uses drugs is to be “seen as a victim, not a criminal” [7, p. 41, ¶ 81]. It therefore is beyond argument that an arrest or prosecution brought for the purpose of bringing an adolescent aged 10–17 who sells sex or uses drugs before a juvenile or criminal court is violative of international law.

The question then becomes whether the non-criminalization principle bars the custodial arrest of adolescents aged 10–17 who use drugs or sell sex for purposes of bringing state custody proceedings against the adolescent for commission of a status offence or an abuse or neglect proceeding. While this question is beyond the scope of this article and will be addressed in a future writing, it is the author's position that custodial arrests are, as a form of temporary detention and by virtue of the involvement of uniformed services, a *per se* violation of the non-criminalization principle contained in international guidance such as the Riyadh Guidelines and the 2010 UN Guidelines on the Alternative Care of Children.

The CRC Committee's jurisprudence also specifically contemplates the health-related dangers of law enforcement-based interventions on adolescent KPs aged 10–17. The Committee's General Comment on HIV/AIDS acknowledges that rape and other sexual abuse by child protection officers, law enforcement and detention personnel expose adolescents to increased risk of sero-conversion [18]. The Committee's General Comment on Adolescent Health and Development recommends comprehensive health services specific to adolescents who sell sex and notes that it is the obligation of States parties to treat such youth “as victims and not as offenders” [22, p. 10, ¶ 37] and that States parties should also “ensure adolescents affected by poverty who are socially marginalised are not criminalised” [22, p. 4, ¶ 12].

Despite the clarity of the Committee's decisions concerning the principle of non-criminalization, its periodic reporting guidelines fail to adequately apprise or require reporting on States parties’ attendance to the health consequences specific to adolescents in conflict with the law. The CRC Committee's periodic reporting mechanism would be improved were it to more strictly account for health consequences affecting adolescents in conflict with the law. While the protection of adolescents from drug use and dependence is now appropriately dealt with under the “disability, basic health and welfare” cluster, the protection of adolescents from sexual exploitation and sexual abuse, children in street situations, and children in conflict with the law improperly remain under the “special protection measures” cluster [23,24]. Under the “disability, basic health and welfare” cluster, States parties are required to take into account the General Comment on HIV/AIDS [23,24]. While the CRC Committee requires that States parties take into account the Committee's jurisprudence on children's rights in juvenile justice under the “special protection measures” cluster, it is rarely the case that States parties do so in the context of adolescents who sell sex or use drugs [23,24].

#### The right to voluntary, confidential and adolescent-friendly health services

It is common for legal analyses of health interventions relevant to KPs aged 10–17 to rely on a “right to health” lens. The reliance on the right to health provisions is fitting for a movement organized around “universal access,” as the Convention clarifies that no adolescent aged 10–17 must be “deprived of his or her right of access to such health care services” [14, art. 24(1)]. Article 24 of the Convention requires that signatories “recognise the right of the child to the enjoyment of the highest attainable standard of health and to facilities for the treatment of illness and rehabilitation of health” [14]. States parties are also obligated “to promote physical and psychological recovery and social reintegration of a child victim of: any form of neglect, exploitation, or abuse …” [14, art. 39]. It is important to note that this principle is to be read in tandem with the Committee's jurisprudence on the principle of non-criminalization such that *involuntary* “rehabilitation” is violative of international law.

The right to health must not be restricted to health and mental health services, but include the article 26 right to social security and article 27 right to a standard of living adequate for adolescent development, particularly clothing, nutrition and, if desired, immediate shelter and long-term housing [1,2,14,25,26]. It is therefore advisable that health practitioners working with adolescents aged 10–17 who use drugs or sell sex integrate comprehensive social services into health programmes, and establish a reliable and safe referral network.

The “health and human rights” framework is also appealed to in support of adolescents’ sexual and reproductive health and rights. The CRC Committee's General Comment No. 15 in particular elaborates on these principles [25]. The Committee stipulates that freedoms inherent in children's right to health “include the right to control one's health and body, including sexual and reproductive freedom to make responsible choices” [25, p. 8, ¶ 24]. The Committee has interpreted this right rather broadly in reference to services, to include a right to access a range of facilities and goods as well as prevention, treatment, rehabilitation and palliative care services [25].

On the other hand, the CRC Committee has been less clear on the parameters of parental consent. The Committee suggests waiver is necessary for life-saving treatment but at the same time attenuates the right to sexual and reproductive health according to the Convention's principle of “evolving capacities,” contained in article 5 of the Convention [14,22]. This principle is also leveraged to support the view that States parties bear responsibility to build adolescents’ capacity for informed decision-making in these matters through comprehensive sexuality education and access to confidential and adolescent-friendly sexual and reproductive health services [15,16]. Those who provide healthcare may also rely on the fact that many forms of sexual and reproductive health services are life-saving in nature, including but not limited to prevention, care and treatment of HIV, access to safe abortion and access to gender-affirming treatment for TG adolescents.

The Convention also firmly guarantees the right to privacy (article 16), particularly in the context of HIV prevention, treatment and care of adolescents [14,18]. The CRC Committee's General Comment on HIV/AIDS states that State parties “must protect the confidentiality of HIV test results … including *within* health and social welfare settings, and information on the HIV status of children may not be disclosed to third parties, including parents, without the child's consent” [18, p. 8, ¶ 24]. Nonetheless, health professionals and other service providers report a conflict between their reporting obligations and the young person's expectation of confidential care [1,2,8]. The CRC Committee has yet to rule definitively on the right to privacy in relation to mandatory reporting, and contrary domestic laws may be in force.

While there already exists powerful guidance on consent and confidentiality for young adult and adult KPs [12,13,27], international actors are cautious in advancing similar recommendations for KPs aged 10–17. The WHO has recommended HIV testing and counselling, with linkages to prevention, treatment and care, for adolescents from KPs in all epidemic scenarios, and specified that consent and confidentiality must be ensured so that services are not used in punitive or coercive ways for adolescent KPs [28]. Yet in still other cases bold and necessary recommendations for rights-based health programming are lacking. For instance, while the UN system endorsed a core package of nine essential harm-reduction services for people who inject drugs which have been shown to reduce HIV infections [13], they are not youth-focused, and key issues regarding young people, IDU and HIV may be falling between the priority areas of different international organizations [29].

It is clear from the Committee's decisions that it not only rejects this trepidation but also argues for the opposite view, namely that the more vulnerable the adolescent, the more critical the right to informed consent and confidentiality. The CRC Committee has specifically held in its General Comment on HIV/AIDS that the Convention requires States parties to ensure access to voluntary, confidential HIV counseling and testing and that prevention programmes must “acknowledge the realities of the lives of adolescents” [18, p. 4, ¶ 11]. The CRC Committee has repeatedly emphasized the importance of adolescent-friendly health services that are “friendly and supportive, provide a wide range of services and information, are geared to their needs, give them the opportunity to participate in decisions affecting their health, are accessible, affordable, confidential and non-judgemental, do not require parental consent and are not discriminatory” [18, p. 7, ¶ 20].

The World Health Organization's quality of care framework provides a useful metric for “adolescent-friendly services” in practice for adolescent KPs: available, accessible, appropriate, equitable and effective [30–32]. In certain locales, health services such as first- or second-line antiretroviral drugs (ARV) or safe abortions are simply not *available* to anyone regardless of age [30,33]. Where health services are available, however, adolescents may yet find them not *accessible* due to unaffordability, remoteness of location or incongruity of hours, or restrictive laws and policies, such as denial of services to non-citizens or migrants, bans on provision of contraception to unmarried adolescents who sell sex, prohibitions on sterile injecting equipment and hormone treatment for adolescents who inject drugs and TG adolescents, or stringent identification requirements [30,33]. Still other services that are available and accessible may yet be delivered in such a way that adolescents are not willing to use them because they are not *acceptable* or safe for young people, for instance, by a doctor known to criticize YMSM who sell sex about the origin of STIs or their feminine appearance, or engage in regular breaches of confidentiality as to the young person's behaviour or HIV status [30,33]. Further, health services must be *appropriate* such that the health services an adolescent actually needs are provided, such as an adolescent who sells sex seeking PrEP and not simply condom distribution or counseling, and *effective* in that the right health services are provided in the right way, and make a positive contribution to the adolescent's health [32]. Finally, health services must be *equitable* to the extent that they do not cater to some adolescent groups and not others, such as a clinic that provides confidential HIV prevention, care and treatment to young people from high-income backgrounds but does not reach street-based young people [30,31]. In other words, all adolescents and not just selected groups are able to obtain the health services that are available [32].

#### The right of meaningful participation in decision-making regarding policy and health programmes

The CRC Committee has identified the article 12 right to be heard as one of the four general principles of the Convention, and repeatedly emphasized it is also to be considered in the interpretation and implementation of all other rights [14,34]. The Committee has specified that this right includes the meaningful participation of adolescents in decision-making, policymaking and preparation of laws, as well as the adoption of complaint procedures and remedies [34]. The CRC Committee's General Comment on HIV/AIDS notes that States parties must provide adolescents with the means “to fully participate at both community and national levels in HIV policy and programme conceptualization, design, implementation, coordination, monitoring and review” [18, p. 5, ¶ 12].

In the context of adolescents aged 10–17 who sell sex or use drugs, in crediting this right it is particularly important that health practitioners consider the greater capacity of adolescents 10–17 who are living independently, have no parents/guardians or no contact with them, have abusive parents/guardians, or who are pregnant [1,2,34].

Article 12 of the International Covenant on Economic, Social and Cultural Rights (ICESCR) further reinforces the right of all people to the highest attainable standard of physical and mental health [35]. The ESCR Committee's General Comment 14 recommends States parties ensure adolescent-friendly healthcare, and adolescents’ “opportunity to participate in decisions affecting their health, to build life-skills, to acquire appropriate information, to receive counselling and to negotiate the health-behaviour choices they make” [36, p. 9, ¶ 23].

#### The right to informed consent and to refuse treatment and research trials

The mixed policy and programme environment has resulted in longstanding limitations on data collection, service provision and medical treatment to adolescent KPs aged 10–17 [1,2,37,38]. The reluctance by international actors to take a position is reflected in the dearth of medical trials, monitoring or evaluation of adolescent KPs aged 10–17 who sell sex or use drugs. Even where surveys do monitor prevalence and trends of drug use among young people, they are almost always still based on school samples that neglect street-based and out-of-school youth, and people who inject drugs remain largely invisible in the official statistics on youth drug use [29].

The “solutions” to the research gap proposed by international health actors remain incomplete as a result of unclear international legal guidance. For instance, in a series of joint UNAIDS and WHO meetings regarding ethical guidelines for engaging PWID in HIV prevention trials, it was recommended that researchers seek the adolescents’ permission to disclose use of injecting drugs before making contact with parents, and if they are not willing to do so, they should not be included in the study [37,38]. While the approach appropriately honours the right to privacy and right to refuse or consent to participation in medical treatment or research trials by preventing disclosure of drug use or the sale of sex to guardians, it fails to confirm the right of adolescents to confidentiality *and* at the same time the positive right to go forward with said life-saving treatment [18]. As a result, service providers may resort to not asking clients their age in order to provide them with assistance, and to avoid enforcement of age restrictions on accessing harm-reduction services, preventing the disaggregation of data by age [7].

#### The right to treatment and waiver of parental consent

Current treatment guidelines fail to honour the right of adolescents to life-saving treatment. In the joint UNAIDS and WHO Eastern Europe & Central Asia experts meetings regarding ethical guidelines for engaging adolescents aged 10–17 who inject drugs in HIV prevention trials, researchers concluded that absent parental consent for medical treatment for HIV or STIs, NSP or OST, researchers may not provide this life-saving treatment to adolescents where parental consent is required by domestic legislation [38]. In the words of the consultation report, “[r]esearchers should not conduct trials with proven interventions with the aim of bringing about change in law and policy” [37, p. 27].

The Committee recently explained that children, in accordance with their evolving capacities, should have access to confidential counselling without consent of a guardian or a parent [14,34]. In addition, states should consider “allowing children to consent to certain medical treatments and interventions without the permission of a parent, caregiver, or guardian, such as HIV testing and sexual and reproductive health services, including education and guidance on sexual health, contraception and safe abortion” [25, p. 9, ¶ 31]. It should go without saying that life-saving medical treatment for HIV or STIs, and critical harm-reduction resources such as NSP and OST, must be made available to all adolescents – whose right to life and health trumps a guardian's right to care and custody. It is incumbent on the Committee on the Rights of the Child to elaborate on these opinions, and make clear that even the most marginalized adolescents are provided life-saving medical treatment.

## Conclusions

The “cure,” as the idiom goes, may be worse than the problem it intends to remedy. This is precisely the case for health interventions among adolescents aged 10–17 who use drugs or sell sex. Without full implementation of the above principles, funders, researchers, health practitioners and community-based KP groups may ignore the urgent needs of KPs aged 10–17 in order to protect themselves from prosecution. Without appropriate guidance, the medical analogue to the principle of minimal intervention – “first, do no harm” – may persuade peer and other health practitioners to practice life-saving interventions in the shadow of threatening and outdated interpretations of the law.

## Author information

Brendan Michael Conner, Esq., is a Staff Attorney at Streetwise and Safe, where he provides civil legal representation and policy support to LGBTQQ youth of color who experience police encounters and government benefits discrimination in New York City, including youth with experience in trading sex for survival needs. He has served as an expert on international law and young key populations, including as Technical Advisor to the HIV Young Leaders Fund. He has worked both independently and in partnership with the Fremont Center on projects for clients such as Safe Horizon's Streetwork Project, the UN Development Programme (UNDP), the United States Agency for International Development (USAID) and the Open Society Institute. The opinions expressed in this article are the author's own and do not necessarily reflect the views of Streetwise and Safe.

## References

[1] WHO, UNAIDS, IAWG (2014). HIV and young people who sell sex: a technical brief.

[2] WHO, UNAIDS, IAWG (2014). HIV and young people who sell drugs: a technical brief.

[3] WHO, UNAIDS, Inter-Agency Working Group on Key Populations (“IAWG”) (2014). HIV and young men who have sex with men: a technical brief.

[4] WHO, UNAIDS, IAWG (2014). HIV and young transgender people: a technical brief. Draft report.

[5] Global Commission on HIV and the Law (2012). HIV and the law: risks, rights and health.

[6] Rahman F Legal and policy considerations in reaching young key populations.

[7] Barrett D, Veerman P (2012). Article 33: protection from narcotic drugs and psychotropic substances.

[8] Conner B, Mago A, Middleton-Lee S (2014). Sexual and reproductive health needs and access to health services for adolescents under 18 engaged in selling sex in Asia Pacific.

[9] Youth Voices Count (2013). Policy brief on self-stigma among young men who have sex with men and young transgender women and the linkages with HIV in Asia.

[10] UNESCO, UNFPA, UNAIDS, UNDP, Youth Lead (2013). Young people and the law in Asia and the Pacific: a review of laws and policies affecting young people's access to sexual and reproductive health and HIV services.

[11] McClure C, Chandler C, Bissell S (2014). Responses to HIV in sexually exploited children or adolescents who sell sex. Lancet.

[12] WHO, UNFPA, UNAIDS, NSWP (2012). Prevention and treatment of HIV and other sexually transmitted infections for sex workers in low-and-middle-income countries: recommendations for a public health approach.

[13] WHO, UNODC, UNAIDS (2009). Technical guide for countries to set targets for universal access to HIV prevention, treatment and care for injecting drug users.

[14] UN Convention on the Rights of the Child, G.A. res. 44/25, annex, 44 UN GAOR Supp. (No. 49) at 167, UN Doc. A/44/49 (1989), entered into force Sept. 2 (1990).

[15] Schabas W, Sax H (2006). Article 37: prohibition of torture, death penalty, life imprisonment and deprivation of liberty.

[16] CRC Committee

[17] CRC Committee

[18] CRC Committee

[19] Optional Protocol to the Convention on the Rights of the Child on the Sale of Children, Child Prostitution and Child Pornography, adopted by G.A. Res. 54/263, opened for signature 25 May 2000 (entered into force 18 January 2002)

[20] Muntarbhorn V (2007). Article 34: sexual exploitation and sexual abuse of children.

[21] Report of the Committee on the Rights of the Child General Assembly, Sixty-third Session.

[22] CRC Committee (2003). General Comment No. 4: adolescent health and development in the context of the Convention on the Rights of the Child CRC/GC/2003/4.

[23] CRC Committee

[24] CRC Committee

[25] CRC Committee

[26] Eide A, Eide B (2006). Article 24: the right to health.

[27] WHO, UNAIDS, MSMGF, UNDP (2011). Prevention and treatment of HIV and other sexually transmitted infections among men who have sex with men and transgender people: recommendations for a public health approach.

[28] WHO Consolidated guidelines on the use of antiretroviral drugs for treating and preventing HIV infection: recommendations for a public health approach.

[29] Fletcher A, Krug A, Stoicescu C (2012). Excluding youth? A global review of harm reduction services for young people. The global state of harm reduction 2012: towards an integrated response.

[30] WHO (2003). Adolescent-friendly health services: an agenda for change.

[31] WHO (2012). Making health services adolescent friendly: developing national quality standards for adolescent friendly health services.

[32] WHO (2009). Quality assessment guidebook. A guide to assessing health services for adolescent clients.

[33] Conner B (Forthcoming). “First, do no harm”: an advocacy brief on sexual and reproductive health needs and access to health services for adolescents 10–17 engaged in selling sex in the Asia Pacific.

[34] General Comment No. 12: right of the child to be heard, UN Doc

[35] International Covenant on Economic

[36] Committee on Economic, Social and Cultural Rights (“CESCR”)

[37] WHO, UNAIDS (2010). Ethical engagement of people who inject drugs in HIV prevention trials.

[38] WHO, UNAIDS (2011). Ethical engagement of people who inject drugs in HIV prevention trials.

